# Supplementary material for: Uncertainty in serious illness: A national interdisciplinary consensus exercise to identify clinical research priorities
Source: PLoS One. 2024 Feb 29;19(2):e0289522. doi: 10.1371/journal.pone.0289522 (PMC10903860; doi:10.1371/journal.pone.0289522)
Supplement: S1 File — (DOCX) [file pone.0289522.s001.docx]

**Supporting information 1: Topic guide for focus groups**

**Facilitator’s introduction**

1. **The facilitator introduces self**
2. **The facilitator checks that all have completed a consent form and demographics questionnaire, remind the participants about audio recording for research purposes and reassures them about confidentiality and anonymity.**
3. **The facilitator briefly explains the purpose of the discussion:** “We are all here because we’re interested in the uncertainties that can occur in serious illness, and how this can affect people with illness and those caring for them. We will all have different views and perspectives on uncertainty and the aim of today’s discussion is to share experiences and build a consensus on the priorities for future research. This focus group is the first step of that consensus process”
4. **The facilitator explains the ground rules for the focus group discussion:**

**Focus group discussion**

**Experiences Ask participants to introduce themselves in turn, and give a short example of how they have experienced a situation of uncertainty concerning serious illness**

**The effects of uncertainty: What effects can uncertainty have in situations of serious illness?**

- Consider physical, practical, psychological, existential, social, and temporal domains.
- Consider effects at individual and system levels.

**When confronted with uncertainty, what should we be aiming for? What outcomes are important?**

- Individual and system-level outcomes
- Outcomes for patients, carers, health professionals

**What are the key research questions and priorities to achieve these outcomes?**

- What situations is it important to investigate uncertainty in?
- What aspects of uncertainty do we need to know more about?
- What aspects of addressing or managing uncertainty are important?

**Summary and list writing: Facilitator (or scribe) writes a list of the items identified in section 4 & asks if there are other areas or items to add to the list.**

Thank participants and reiterate confidentiality.

**Item list: (facilitator to list all research questions and priorities identified during the focus group discussion)**

…………………………………………………………………. ………………………………………………………………….

…………………………………………………………………. ………………………………………………………………….

…………………………………………………………………. ………………………………………………………………….

…………………………………………………………………. ………………………………………………………………….

…………………………………………………………………. ………………………………………………………………….

…………………………………………………………………. ………………………………………………………………….

…………………………………………………………………. ………………………………………………………………….

…………………………………………………………………. ………………………………………………………………….

…………………………………………………………………. ………………………………………………………………….

…………………………………………………………………. ………………………………………………………………….

…………………………………………………………………. ………………………………………………………………….

…………………………………………………………………. ………………………………………………………………….

**Facilitator notes:**Please write a short (1-2 paragraph) reflection below about how you felt the session went as soon as possible afterwards and return it to [researcher name] You may wish to comment on group dynamics or the tone/mood of the session, as well as how you felt as the facilitator.

**Facilitator reflection:**

**Facilitator name:**

**Group number:**
