# Supplementary material for: Uncertainty in serious illness: A national interdisciplinary consensus exercise to identify clinical research priorities
Source: PLoS One. 2024 Feb 29;19(2):e0289522. doi: 10.1371/journal.pone.0289522 (PMC10903860; doi:10.1371/journal.pone.0289522)
Supplement: S2 File — (DOCX) [file pone.0289522.s002.docx]

**Supporting information 2: Workshop Schedule**

| **Time** | **Session** |
| --- | --- |
| **09.30** | **Arrival and coffee** |
| **10.00 – 10:10** | Welcome and intro |
| **10:10 – 10.50** | Introduction to uncertainty – why is it so important?  Models of uncertainty, what do we know? |
| **10.50 – 11.00** | Q&A with speakers |
| **11:00 – 11:10** | Set up for focus groups |
| **11:10 – 12:10** | CERTAIN Study focus groups |
| **12:10 – 13:00** | **Lunch** |
| **13:00 – 14:00** | Interventions to address uncertainty  Case study: implementing the SPACE toolkit  Communicating uncertainty: the OSCAR study |
| **14.00 – 14.15** | Q&A with speakers |
| **14:15 – 14.30** | **Break** |
| **14.30 – 15.15** | Presentation of priority list, ranking exercise and results |
| **15.15** | Reflections and Conclusion |
