# Supplementary material for: Uncertainty in serious illness: A national interdisciplinary consensus exercise to identify clinical research priorities
Source: PLoS One. 2024 Feb 29;19(2):e0289522. doi: 10.1371/journal.pone.0289522 (PMC10903860; doi:10.1371/journal.pone.0289522)
Supplement: S4 File — (DOCX) [file pone.0289522.s004.docx]

**Supporting information 4: Full quantitative dataset from ranking exercise**

| Poll Type | Poll Question | Poll Option | Score |
| --- | --- | --- | --- |
| Ranking | Please rank the top 3 research priorities relating to serious illness uncertainty from this list | Communication of uncertainty | 3 |
| Ranking | Please rank the top 3 research priorities relating to serious illness uncertainty from this list | Communication of uncertainty | 3 |
| Ranking | Please rank the top 3 research priorities relating to serious illness uncertainty from this list | Communication of uncertainty | 3 |
| Ranking | Please rank the top 3 research priorities relating to serious illness uncertainty from this list | Communication of uncertainty | 3 |
| Ranking | Please rank the top 3 research priorities relating to serious illness uncertainty from this list | Communication of uncertainty | 3 |
| Ranking | Please rank the top 3 research priorities relating to serious illness uncertainty from this list | Communication of uncertainty | 3 |
| Ranking | Please rank the top 3 research priorities relating to serious illness uncertainty from this list | Communication of uncertainty | 3 |
| Ranking | Please rank the top 3 research priorities relating to serious illness uncertainty from this list | Communication of uncertainty | 3 |
| Ranking | Please rank the top 3 research priorities relating to serious illness uncertainty from this list | Communication of uncertainty | 3 |
| Ranking | Please rank the top 3 research priorities relating to serious illness uncertainty from this list | Communication of uncertainty | 3 |
| Ranking | Please rank the top 3 research priorities relating to serious illness uncertainty from this list | Communication of uncertainty | 3 |
| Ranking | Please rank the top 3 research priorities relating to serious illness uncertainty from this list | Communication of uncertainty | 3 |
| Ranking | Please rank the top 3 research priorities relating to serious illness uncertainty from this list | Communication of uncertainty | 3 |
| Ranking | Please rank the top 3 research priorities relating to serious illness uncertainty from this list | Communication of uncertainty | 3 |
| Ranking | Please rank the top 3 research priorities relating to serious illness uncertainty from this list | Communication of uncertainty | 3 |
| Ranking | Please rank the top 3 research priorities relating to serious illness uncertainty from this list | Education/training of HCPs | 3 |
| Ranking | Please rank the top 3 research priorities relating to serious illness uncertainty from this list | Education/training of HCPs | 3 |
| Ranking | Please rank the top 3 research priorities relating to serious illness uncertainty from this list | Explore positive aspects of uncertainty | 3 |
| Ranking | Please rank the top 3 research priorities relating to serious illness uncertainty from this list | How best to manage/approach uncertainty | 3 |
| Ranking | Please rank the top 3 research priorities relating to serious illness uncertainty from this list | How best to manage/approach uncertainty | 3 |
| Ranking | Please rank the top 3 research priorities relating to serious illness uncertainty from this list | How best to manage/approach uncertainty | 3 |
| Ranking | Please rank the top 3 research priorities relating to serious illness uncertainty from this list | How best to manage/approach uncertainty | 3 |
| Ranking | Please rank the top 3 research priorities relating to serious illness uncertainty from this list | How best to manage/approach uncertainty | 3 |
| Ranking | Please rank the top 3 research priorities relating to serious illness uncertainty from this list | How best to manage/approach uncertainty | 3 |
| Ranking | Please rank the top 3 research priorities relating to serious illness uncertainty from this list | How to cope with uncertainty (including how to empower people to manage their own uncertainty) | 3 |
| Ranking | Please rank the top 3 research priorities relating to serious illness uncertainty from this list | How to cope with uncertainty (including how to empower people to manage their own uncertainty) | 3 |
| Ranking | Please rank the top 3 research priorities relating to serious illness uncertainty from this list | How to cope with uncertainty (including how to empower people to manage their own uncertainty) | 3 |
| Ranking | Please rank the top 3 research priorities relating to serious illness uncertainty from this list | How to cope with uncertainty (including how to empower people to manage their own uncertainty) | 3 |
| Ranking | Please rank the top 3 research priorities relating to serious illness uncertainty from this list | How to cope with uncertainty (including how to empower people to manage their own uncertainty) | 3 |
| Ranking | Please rank the top 3 research priorities relating to serious illness uncertainty from this list | How to cope with uncertainty (including how to empower people to manage their own uncertainty) | 3 |
| Ranking | Please rank the top 3 research priorities relating to serious illness uncertainty from this list | Impact of uncertainty on bereavement | 3 |
| Ranking | Please rank the top 3 research priorities relating to serious illness uncertainty from this list | Understanding patient/carer experiences of uncertainty in depth | 3 |
| Ranking | Please rank the top 3 research priorities relating to serious illness uncertainty from this list | Understanding patient/carer experiences of uncertainty in depth | 3 |
| Ranking | Please rank the top 3 research priorities relating to serious illness uncertainty from this list | Variation in experience/response to uncertainty between different individuals, groups, professions | 3 |
| Ranking | Please rank the top 3 research priorities relating to serious illness uncertainty from this list | Communication of uncertainty | 2 |
| Ranking | Please rank the top 3 research priorities relating to serious illness uncertainty from this list | Communication of uncertainty | 2 |
| Ranking | Please rank the top 3 research priorities relating to serious illness uncertainty from this list | Communication of uncertainty | 2 |
| Ranking | Please rank the top 3 research priorities relating to serious illness uncertainty from this list | Education/training of HCPs | 2 |
| Ranking | Please rank the top 3 research priorities relating to serious illness uncertainty from this list | Education/training of HCPs | 2 |
| Ranking | Please rank the top 3 research priorities relating to serious illness uncertainty from this list | Education/training of HCPs | 2 |
| Ranking | Please rank the top 3 research priorities relating to serious illness uncertainty from this list | Education/training of HCPs | 2 |
| Ranking | Please rank the top 3 research priorities relating to serious illness uncertainty from this list | Education/training of HCPs | 2 |
| Ranking | Please rank the top 3 research priorities relating to serious illness uncertainty from this list | Education/training of HCPs | 2 |
| Ranking | Please rank the top 3 research priorities relating to serious illness uncertainty from this list | Education/training of HCPs | 2 |
| Ranking | Please rank the top 3 research priorities relating to serious illness uncertainty from this list | Explore positive aspects of uncertainty | 2 |
| Ranking | Please rank the top 3 research priorities relating to serious illness uncertainty from this list | How best to manage/approach uncertainty | 2 |
| Ranking | Please rank the top 3 research priorities relating to serious illness uncertainty from this list | How best to manage/approach uncertainty | 2 |
| Ranking | Please rank the top 3 research priorities relating to serious illness uncertainty from this list | How to cope with uncertainty (including how to empower people to manage their own uncertainty) | 2 |
| Ranking | Please rank the top 3 research priorities relating to serious illness uncertainty from this list | How to cope with uncertainty (including how to empower people to manage their own uncertainty) | 2 |
| Ranking | Please rank the top 3 research priorities relating to serious illness uncertainty from this list | How to cope with uncertainty (including how to empower people to manage their own uncertainty) | 2 |
| Ranking | Please rank the top 3 research priorities relating to serious illness uncertainty from this list | How to cope with uncertainty (including how to empower people to manage their own uncertainty) | 2 |
| Ranking | Please rank the top 3 research priorities relating to serious illness uncertainty from this list | How to cope with uncertainty (including how to empower people to manage their own uncertainty) | 2 |
| Ranking | Please rank the top 3 research priorities relating to serious illness uncertainty from this list | Impact of uncertainty on bereavement | 2 |
| Ranking | Please rank the top 3 research priorities relating to serious illness uncertainty from this list | Impact of uncertainty on bereavement | 2 |
| Ranking | Please rank the top 3 research priorities relating to serious illness uncertainty from this list | Uncertainty in specific conditions/clinical situations | 2 |
| Ranking | Please rank the top 3 research priorities relating to serious illness uncertainty from this list | Uncertainty in specific conditions/clinical situations | 2 |
| Ranking | Please rank the top 3 research priorities relating to serious illness uncertainty from this list | Uncertainty in specific conditions/clinical situations | 2 |
| Ranking | Please rank the top 3 research priorities relating to serious illness uncertainty from this list | Understanding patient/carer experiences of uncertainty in depth | 2 |
| Ranking | Please rank the top 3 research priorities relating to serious illness uncertainty from this list | Understanding patient/carer experiences of uncertainty in depth | 2 |
| Ranking | Please rank the top 3 research priorities relating to serious illness uncertainty from this list | Understanding patient/carer experiences of uncertainty in depth | 2 |
| Ranking | Please rank the top 3 research priorities relating to serious illness uncertainty from this list | Understanding patient/carer experiences of uncertainty in depth | 2 |
| Ranking | Please rank the top 3 research priorities relating to serious illness uncertainty from this list | Understanding patient/carer experiences of uncertainty in depth | 2 |
| Ranking | Please rank the top 3 research priorities relating to serious illness uncertainty from this list | Understanding patient/carer experiences of uncertainty in depth | 2 |
| Ranking | Please rank the top 3 research priorities relating to serious illness uncertainty from this list | Understanding patient/carer experiences of uncertainty in depth | 2 |
| Ranking | Please rank the top 3 research priorities relating to serious illness uncertainty from this list | Variation in experience/response to uncertainty between different individuals, groups, professions | 2 |
| Ranking | Please rank the top 3 research priorities relating to serious illness uncertainty from this list | Variation in experience/response to uncertainty between different individuals, groups, professions | 2 |
| Ranking | Please rank the top 3 research priorities relating to serious illness uncertainty from this list | Variation in experience/response to uncertainty between different individuals, groups, professions | 2 |
| Ranking | Please rank the top 3 research priorities relating to serious illness uncertainty from this list | Variation in experience/response to uncertainty between different individuals, groups, professions | 2 |
| Ranking | Please rank the top 3 research priorities relating to serious illness uncertainty from this list | Communication of uncertainty | 1 |
| Ranking | Please rank the top 3 research priorities relating to serious illness uncertainty from this list | Communication of uncertainty | 1 |
| Ranking | Please rank the top 3 research priorities relating to serious illness uncertainty from this list | Communication of uncertainty | 1 |
| Ranking | Please rank the top 3 research priorities relating to serious illness uncertainty from this list | Education/training of HCPs | 1 |
| Ranking | Please rank the top 3 research priorities relating to serious illness uncertainty from this list | Education/training of HCPs | 1 |
| Ranking | Please rank the top 3 research priorities relating to serious illness uncertainty from this list | Education/training of HCPs | 1 |
| Ranking | Please rank the top 3 research priorities relating to serious illness uncertainty from this list | Education/training of HCPs | 1 |
| Ranking | Please rank the top 3 research priorities relating to serious illness uncertainty from this list | Education/training of HCPs | 1 |
| Ranking | Please rank the top 3 research priorities relating to serious illness uncertainty from this list | Education/training of HCPs | 1 |
| Ranking | Please rank the top 3 research priorities relating to serious illness uncertainty from this list | Education/training of HCPs | 1 |
| Ranking | Please rank the top 3 research priorities relating to serious illness uncertainty from this list | Education/training of HCPs | 1 |
| Ranking | Please rank the top 3 research priorities relating to serious illness uncertainty from this list | Education/training of HCPs | 1 |
| Ranking | Please rank the top 3 research priorities relating to serious illness uncertainty from this list | Explore positive aspects of uncertainty | 1 |
| Ranking | Please rank the top 3 research priorities relating to serious illness uncertainty from this list | Explore positive aspects of uncertainty | 1 |
| Ranking | Please rank the top 3 research priorities relating to serious illness uncertainty from this list | Explore positive aspects of uncertainty | 1 |
| Ranking | Please rank the top 3 research priorities relating to serious illness uncertainty from this list | Explore positive aspects of uncertainty | 1 |
| Ranking | Please rank the top 3 research priorities relating to serious illness uncertainty from this list | Factors associated with different uncertainty experiences | 1 |
| Ranking | Please rank the top 3 research priorities relating to serious illness uncertainty from this list | Factors associated with different uncertainty experiences | 1 |
| Ranking | Please rank the top 3 research priorities relating to serious illness uncertainty from this list | Factors associated with different uncertainty experiences | 1 |
| Ranking | Please rank the top 3 research priorities relating to serious illness uncertainty from this list | How best to manage/approach uncertainty | 1 |
| Ranking | Please rank the top 3 research priorities relating to serious illness uncertainty from this list | How best to manage/approach uncertainty | 1 |
| Ranking | Please rank the top 3 research priorities relating to serious illness uncertainty from this list | How best to manage/approach uncertainty | 1 |
| Ranking | Please rank the top 3 research priorities relating to serious illness uncertainty from this list | How best to manage/approach uncertainty | 1 |
| Ranking | Please rank the top 3 research priorities relating to serious illness uncertainty from this list | How to cope with uncertainty (including how to empower people to manage their own uncertainty) | 1 |
| Ranking | Please rank the top 3 research priorities relating to serious illness uncertainty from this list | How to cope with uncertainty (including how to empower people to manage their own uncertainty) | 1 |
| Ranking | Please rank the top 3 research priorities relating to serious illness uncertainty from this list | How to cope with uncertainty (including how to empower people to manage their own uncertainty) | 1 |
| Ranking | Please rank the top 3 research priorities relating to serious illness uncertainty from this list | Impact of uncertainty on bereavement | 1 |
| Ranking | Please rank the top 3 research priorities relating to serious illness uncertainty from this list | Uncertainty in specific conditions/clinical situations | 1 |
| Ranking | Please rank the top 3 research priorities relating to serious illness uncertainty from this list | Understanding patient/carer experiences of uncertainty in depth | 1 |
| Ranking | Please rank the top 3 research priorities relating to serious illness uncertainty from this list | Understanding patient/carer experiences of uncertainty in depth | 1 |
| Ranking | Please rank the top 3 research priorities relating to serious illness uncertainty from this list | Understanding patient/carer experiences of uncertainty in depth | 1 |
| Ranking | Please rank the top 3 research priorities relating to serious illness uncertainty from this list | Understanding patient/carer experiences of uncertainty in depth | 1 |
| Ranking | Please rank the top 3 research priorities relating to serious illness uncertainty from this list | Understanding patient/carer experiences of uncertainty in depth | 1 |
| Ranking | Please rank the top 3 research priorities relating to serious illness uncertainty from this list | Variation in experience/response to uncertainty between different individuals, groups, professions | 1 |
